# Whole genome bisulfite sequencing of *Medicago truncatula* A17 wild type and *lss* mutants

**DOI:** 10.1186/s13104-020-05012-6

**Published:** 2020-03-31

**Authors:** Nowlan H. Freese, Elise L. Schnabel, Julia A. Frugoli

**Affiliations:** 1 Department of Bioinformatics and Genomics, University of North Carolina at Charlotte, Kannapolis, NC 28081 USA; 2 Department of Genetics & Biochemistry, Clemson University, Clemson, SC 29634 USA

**Keywords:** Methylation, Nodulation, *Medicago truncatula*, Autoregulation of nodulation, *lss*

## Abstract

**Objectives:**

Earlier work in our lab identified a spontaneous mutant (*likesunnsupernodulator*-*ls*s) in *Medicago truncatula,* resulting in increased nodulation. Molecular genetic evidence indicated the phenotype was due to an unknown lesion resulting in *cis*-silencing of the *SUNN* gene. Altered methylation of the promoter was suspected, but analysis of the *SUNN* promoter by bisulfite sequencing at the time of publication revealed no significant methylation differences between the *SUNN* promoter in wild type and *lss* plants. Using advances in methylome generation we compared the methylome of wild type and the *lss* mutant in the larger 810 kB area of the genome where *lss* maps.

**Data description:**

The data show the distribution of types of methylation across the entire genome between A17 wild type and *lss* mutants, the number of differentially methylated cytosines between genotypes, and the overall pattern of gene methylation between genotypes. We expect the wild type data will be especially useful as a reference for other investigations of methylation using *M. truncatula*.

## Objective

Legume plants regulate the number of nodules formed through a long distance signal transduction pathway that involves many genes [1]. Earlier work in our lab identified a spontaneous mutant in *Medicago truncatula* in the Jemalong cultivar, resulting in increased nodulation [2]. Molecular genetic evidence indicated the phenotype of the *like*-*sunn*-*supernodulator* (*lss*) mutant was due to an unknown lesion resulting in *cis*-silencing of the *SUNN* gene, which has a wild type sequence in *lss* mutants. Expression of *SUNN* in the shoots is critical to regulation of nodulation in the roots [3]. The lesion is mapped to an 810 kilobase area of the genome on chromosome 4, including the *SUNN* gene, but the nature of the lesion has not been determined [2]. Altered methylation of the promoter was suspected, but analysis of the *SUNN* promoter by bisulfite sequencing at the time of publication revealed no significant methylation differences between the *SUNN* promoter in A17 wild type and *lss* plants [2]. Using genome capture combined with bi-sulfite sequencing, Satgé et al. [4] identified 474 regions that were differentially methylated during nodule development in *M. truncatula* and over 400 genes downregulated in plants with a mutant copy of the *DEMETER* demethylase gene. Because the *lss* lesion behaves like a paramutation, including reversion events [2], we decided to expand our bisulfite sequencing beyond the *SUNN* promoter and compare the methylomes of the A17 wild type to the *lss* mutant.

## Data description

### Whole genome bisulfite sequencing

Data consist of sequencing results of two bisulfite libraries made from young leaves of individual 6-week-old greenhouse grown *Medicago truncatula* A17 wild type and *lss* mutant plants (one of each). DNA was extracted using the DNeasy Plant Mini Kit (Qiagen). Bisulfite treatment of A17 and *lss* DNA was performed using the EZ DNA Methylation-Gold Kit (Zymo Research) according to manufacturer instructions. Whole genome bisulfite sequencing (WGBS) libraries were prepared using the Illumina TruSeq DNA Methylation library preparation kit. Library quality was assessed using an Agilent Bioanalyzer and quantitated using a Q-PCR kit from KAPA Biosystems following the manufacturer’s instructions. Sequencing was performed on an Illumina HiSeq 2500 with the HiSeq Cluster Kit v4 (1 × 125 bp single-read; see Data set 1 and Data set 2 for unaligned sequencing reads). Raw sequencing FASTQ files were trimmed to remove adapters, low-quality bases, and short reads using Trim Galore! v0.4.2 (Quality Phred score cutoff 5, min length 20). Alignment was carried out using Bismark v0.16.3 [5] and Bowtie2 v2.2.9 with N-1 for increased sensitivity against the *M. truncatula* 4.0 genome. Duplicated sequences were removed with Picard MarkDuplicates v2.8.0 and alignments with MAPQ scores of 0 removed with Samtools v1.3.1. Sequencing and alignment results are summarized in data file 1 referenced in Table 1.

**Table 1:** Overview of data files/data sets

Label	Name of data file/data set	File types (file extension)	Data repository and identifier (DOI or accession number)
Data file 1 [9]	Table summarizing WGBS alignment	(.pdf)	10.17605/OSF.IO/B9CKM
Data file 2 [9]	DMC data from A17 and *lss* samples (CG and CHG)	MS Excel file (.xlsx)	10.17605/OSF.IO/B9CKM
Data file 3 [9]	DMR data from A17 and *lss* samples (CG and CHG) including nearest gene to DMR	MS Excel file (.xlsx)	10.17605/OSF.IO/B9CKM
Data file 4 [9]	Visualization of genome wide differential methylation results	(.pdf)	10.17605/OSF.IO/B9CKM
Data file 5 [9]	Visualization of differential methylation of Medtr4g070970	(.pdf)	10.17605/OSF.IO/B9CKM
Data set 1 [10, 11]	A17 sequence data	Sequence file (.fastq)	SRA accession number (fastq.gz): https://identifiers.org/insdc.sra:SRX4457708
Data set 2 [10, 12]	*lss* sequence data	Sequence file (.fastq)	SRA accession number (fastq.gz): https://identifiers.org/insdc.sra:SRX4457709

### Methylation analysis

Methylation calling was performed using MethylDackel v0.2.1. Statistically significant differentially methylated cytosines (DMCs) were identified using the Bioconductor R package DSS v2.14.0 [6] (p.threshold 1e−5) (Data file 2 referenced in Table 1). Differentially methylated regions (DMRs) were also identified through DSS (pct.sig 0.5, minimum number of DMCs 10, 50 bp minimum length, and DMRs merged if within 100 bp) (Data file 3 referenced in Table 1). There were 307 DMRs in the CG context and 772 DMRs in the CHG context. Displays of the number of DMCs in CG and CHG contexts, the number of DMRs identified by comparison of A17 and *lss* in CHG and CG contexts and the distributions of methylation across the exons and 1-kb flanking sequences for A17 and *lss* are graphically displayed in Data file 4 referenced in Table 1. Scripts used to analyze data and produce figures can be found at https://bitbucket.org/nfreese/medicagobseq. Bedtools v2.26.0 was used to associate DMRs with overlapping or closest gene (against *M. truncatula* MedtrA17_4.0 annotation) (Data file 3 referenced in Table 1) [7]. Methylation data was visualized using the Integrated Genome Browser in the area around the receptor protein kinase *SUNN* (Medtr4g070970) gene (Data file 5 referenced in Table 1) [8]. There was a single significant CG DMR within the first Medtr4g070970 exon. No DMRs were identified upstream or downstream within the CG or CHG context. The CG DMR identified within Medtr4g070970 displayed decreased methylation in the A17 sample.

## Limitations

The data sets were generated without biological replicates and thus any comparison of the A17 and *lss* data is limited by a small sample size.

## Data Availability

The data described in this Data note can be freely and openly accessed in the Sequence Read Archive (SRA) at https://identifiers.org/insdc.sra:SRP155259 [10]. Please see Table 1 and reference list for details and links to the data.

## Abbreviations

DMRs: Differentially methylated regions
DMCs: Differentially methylated cytosines
*lss**l**ike*-*s**unn*: *s* *upernodulator*
WGBS: Whole genome bisulfite sequencing
